# Condom use and incarceration among STI clinic attendees in the Deep South

**DOI:** 10.1186/s12889-016-3590-z

**Published:** 2016-09-13

**Authors:** Lauren Brinkley-Rubinstein, Sharon Parker, Annie Gjelsvik, Leandro Mena, Philip A. Chan, Julia Harvey, Brandon Marshall, Curt G. Beckwith, Jennifer Rose, Reginald Riggins, Trisha Arnold, Amy Nunn

**Affiliations:** 1 Department of Social Medicine, University of North Carolina, 341B MacNider Hall, Chapel Hill, NC 27599 USA; 2 Center for Health Equity Research, University of North Carolina, Chapel Hill, NC USA; 3 Department of Sociology and Social Work, North Carolina Agriculture and Technology State University, Greensboro, NC USA; 4 Department of Epidemiology, School of Public Health, Brown University, Providence, RI USA; 5 Department of Medicine, The University of Mississippi Medical Center, Jackson, MS USA; 6 Warren Alpert Medical School of Brown University, Providence, RI USA; 7 The Miriam Hospital, Providence, RI USA; 8 Institute for Community Health Promotion, School of Public Health, Brown University, Providence, RI USA; 9 Department of Psychology, Wesleyan University, Middletown, CT USA

## Abstract

**Background:**

Incarceration history is associated with lower rates of condom use and increased HIV risk. Less is known about duration of incarceration and multiple incarcerations’ impact on condom use post-release.

**Methods:**

In the current study, we surveyed 1,416 adults in Mississippi about their incarceration history and sexual risk behaviors. Generalized estimating equations (GEE) were used to test associations between duration of incarceration, multiple incarcerations, socio-demographic factors, substance use, sexual behavior, and event level condom use at last sex.

**Results:**

After adjusting for covariates, having been incarcerated for at least 6 months two or more times remained significantly associated with condomless sex.

**Conclusions:**

This study found a strong, independent relationship between condom use and multiple, long-term incarceration events among patients in an urban STI clinic in the Deep South. The results suggest that duration of incarceration and multiple incarcerations have significant effects on sexual risk behaviors, underscoring the deleterious impact of long prison or jail sentences on population health. Our findings also suggest that correctional health care professionals and post-release providers might consider offering comprehensive sexual and reproductive health services and those providing community care should consider screening for previous incarceration as a marker of risk.

## Background

Nearly seven million people pass through the criminal justice system in the United States (US) annually, including almost two million in jails or prisons and more than four and a half million on probation or parole [1]. Although the rates of incarceration have gradually declined in recent years, the US continues to have the world’s highest incarceration rates [1].

Incarcerated populations experience higher rates of chronic and infectious diseases than the general populace [2]. Most incarcerated individuals have a history of substance abuse and half qualify as dependent [3–5]. Hepatitis C, HIV, and other sexually transmitted infections (STIs) are highly prevalent in incarcerated populations [6], with rates of HIV for incarcerated individuals three times that of their non-incarcerated counterparts [7]. An estimated 14 % of all HIV positive people in the US pass through a jail at some point in any given year [8].

In Mississippi the rate of HIV is also high. Jackson, Mississippi, has the 4^th^ highest HIV infection rate in the nation at 36.7 per 100,000 in 2011 [9]. The HIV rate among incarcerated populations in Mississippi is 2.2 % for male prisoners and 2.6 % for female prisoners compared to national HIV rates among incarcerated men and women of 1.4 %, 1.9 %, respectively [7].

While a large body of research has focused on the shared risk factors for both incarceration and HIV [10, 11], some previous investigations have specifically evaluated the association between history of incarceration and risky sexual behavior, including condom use [12, 13]. A study in Vancouver, Canada found that among people who inject drugs, those who experienced incarceration in the past 6 months were significantly more likely to report inconsistent condom use with casual partners [14]. Another study that included 106 recently incarcerated men found that 40 % reported multiple sex partners and many reported engaging in condomless sex with committed and casual partners (71 and 26 %, respectively) post-release [15]. Similarly, a recent investigation of 293 African Americans also demonstrated that incarceration was associated with number of sex partners and frequency of condomless sex [16]. Also, Grinstead and colleagues (2008) found that partners of incarcerated men cited inconsistent condom use post-release as a concern [13]. Motivation to engage in condomless sex may increase post-release due to the desire to reconnect with partners, to demonstrate trustworthiness, or for family planning reasons (e.g. intentional pregnancy) [17]. Recent research has also suggested that sexual risk behavior might also increase post-release because of disruption of primary partnerships following incarceration [18]. Finally, criminal justice involvement can create instability in which there is a disconnection from social networks and a lack of housing and employment opportunities all of which have been demonstrated to increase risk-taking behavior [11].

Less is understood, though, about the duration of incarceration and repeated incarceration, and sexual risk behavior. Khan and colleagues (2011) found that HIV infection was associated with both incarceration for 1 year or less and incarceration for 1 year or longer [19]. Building on these findings, the current study aims to understand if repeated incarceration or specific lengths of incarceration (e.g. serving shorter vs. longer sentences) affects sexual risk behavior to varying degrees. This is important to discern because understanding the specific effect of duration of sentence and repeated incarceration could provide essential information about critical points of intervention to lower the risk of condomless sex post-release. This study examines associations between participants’ incarceration experiences, specifically frequency and length of incarceration on condom use at last sex (hereafter “condomless sex”) among STI clinic attendees in Jackson, Mississippi.

## Methods

### Research design

A total of 1,542 participants were enrolled in the study between January and June 2011. All participants were recruited at the Crossroads Clinic, a publicly funded STI clinic in Jackson, Mississippi. Eligibility criteria included: 1) being at least 18 years of age, 2) presenting for STI/HIV screening, 3) willingness to complete a 30-min computerized survey, and 4) ability to speak and read English. All patients presenting at Crossroads Clinic during the study period were offered an opportunity to participate by clinic staff; 93 % of those who were invited to participate completed the survey. Data was collected on a desktop computer with a self-administered survey programmed using *Illume* ™ (*Datstat*, Washington). The survey included questions about demographic, behavioral, structural and social factors. Participants did not receive compensation for their participation. All participants provided informed consent, and the University of Mississippi Medical Center, the Mississippi State Department of Health, and The Miriam Hospital’s institutional review boards approved the study. Of the 1,542 participants, 126 (8.2 %) did not report partner information. Therefore, the final sample included 1,416 participants who reported on at least one sexual partner. These participants reported on condom use for a total of 2,822 sexual events.

### Measures

#### Socio-demographic factors

Socio-demographic information collected included gender, age, sexual orientation, level of education completed, employment status, marital status, housing status, and monthly religious service attendance.

#### Incarceration history

Participants were asked to report the discrete number of times they had ever been incarcerated (e.g. in your lifetime, have you ever been incarcerated (in jail or prison); how many times have you been incarcerated for 6 months or more?) for: 1) 30 days or less, 2) 6 months or less, and 3) more than 6 months. Responses to each question were coded as never, once, or two or more times. These were not mutually exclusive variables. For example, a participant might have been incarcerated for less than 30 days, but also incarcerated another time for more than 6 months. From these variables, a single incarceration experience variable was created with five *mutually exclusive* categories representing never incarcerated, incarcerated only one time for less than 6 months, incarcerated more than one time for less than 6 months, incarcerated only one time for greater than 6 months, and incarcerated more than one time for more than 6 months. Participants also indicated whether their partners had been incarcerated at any time during the past 6 months.

#### Sexual behavior and partner variables

Information related to sexual partnership was also collected. Individuals were asked to list their three most recent sexual partners in the past year. For each of these three partners participants were also asked to: 1) indicate whether or not they considered them to be a main partner (versus a causal partner), 2) state whether or not they (or the partners) were engaging in multiple partnerships at the same time, 3) whether they depended on them (and if these partners depended on the participant) for financial or material resources, including, bills and housing, food, childcare, and transportation costs, 4) if they had used drugs at last sexual encounter, 5) whether they had ever had anal, oral or vaginal sex, and 6) whether they had engaged in condomless sex. We also asked participants to report their total number of lifetime partners.

#### Substance use

Heavy episodic drinking (having more than five alcoholic drinks at one time) and participant or partner alcohol use at last sex for the most recent three sexual partners was measured [20]. Drug measures included frequency and history of marijuana, cocaine and/or crack, and an “other drug use” variable, which measured any use of heroin, methamphetamine, ecstasy, special-k and/or non-prescribed prescription drug use ever.

### Data analysis

Generalized estimating equations (GEE) models were used to test associations between incarceration history, socio-demographic factors, substance use, sexual behavior and event-level condomless sex. GEE analysis was used to account for the fact that participants reported event level data on condom use at last sex with up to three recent sexual partners, leading to correlated outcomes within individuals. Specifically, GEE analysis uses the empirical variance “sandwich” estimator to obtain correctly specified standard errors when the outcomes are correlated [21]. The binary condom use outcome was modeled using a logit link function, and an unstructured within-person correlation matrix was specified. All GEE analyses were conducted using SAS PROC GENMOD (Version 9.2; SAS Institute, 2009).

As a first step, bivariable GEE models were tested for associations between each of the incarceration variables (frequency of and repeated incarceration for < 6 months, and > 6 months), other covariates of interest, and condomless sex. Covariates that were found to be significant at *p* < 0.10 were then retained in a final multivariable GEE logit model. In *post-hoc* analyses, we also tested for interactions between gender and each incarceration variable, and the main partner designation and each incarceration variable. The results showed no significant interactions between gender, main partner designation and the incarceration variables. Therefore, results are provided for the model without interactions.

## Results

The population was largely African American (*N* = 1,343; 94.9 %), and the majority were women (62.6 %, *n* = 884). Participants ranged in age from 18 to 56 years, with a mean age of 24.8 years (SD = 6.13). Nearly six percent (5.7 %) of the population self-identified as bisexual (*n* = 81) and 5.2 % self-identified as gay/lesbian (*n* = 74). More than half (58.6 %) reported having at least some college education (*n* = 828), 52.6 % worked at least part time (*n* = 745), 47.7 % had stable housing (*n* = 675), 35.4 % reported that they financially or materially depended on at least one partner (*n* = 496), and 35.7 % of participants had at least one partner who was dependent on them (*n* = 500). Nineteen percent (19.0 %) of participants reported a history of incarceration (*n* = 262), 17.8 % had been incarcerated for 6 months or less (*n* = 253), 6.0 % had been incarcerated for more than 6 months (*n* = 82), and 6.6 % reported that they had a partner who had been incarcerated in the past six months (*n* = 90). Overall, 11.1 % of participants reported being incarcerated more than once (*n* = 153). Of the 2,822 sexual events, condoms were reportedly used in 45.0 % (*n* = 1,271) of the events. See Table 1 for descriptive statistics.

**Table 1:** Sociodemographic and behavioral characteristics reported by participants attending a publicly funded STI clinic in Jackson, Mississippi (*N* = 1,416)

Variable	N (%)
Female	884 (62.6)
African American	1343 (94.9)
Single	1235 (87.0)
Mean Age (SD, range)	24.8 (6.13, 18–56)
Education	
Some high school	162 (11.5)
High school degree/GED	424 (30.0)
At least some college	828 (58.6)
Currently enrolled in school	611 (43.6)
Currently has stable housing	675 (47.7)
Monthly Income	
< $500	441 (31.5)
$501 – $1,500	518 (37.0)
$1,501 – $3000	264 (18.9)
> $3000	177 (12.6)
Work at least part time	745 (52.6)
Receives at least 1 form of public assistance	579 (41.0)
Financially or materially depend on at least 1 partner	496 (35.4)
At least 1 partner depends on participant	500 (35.7)
Religious Denomination	
Baptist	524 (65.0)
Non-denominational	109 (13.5)
Church of God in Christ	66 (8.2)
Other	33 (13.3)
Attend religious services	
Never	144 (10.5)
Less than a few times per year	172 (12.6)
A few times a year	292 (21.4)
Once or twice a month	312 (22.8)
Almost every week	218 (15.9)
One or more times per week	226 (16.8)
Self-identified sexual orientation	
Heterosexual	1259 (89.0)
Gay/Lesbian	74 (5.2)
Bisexual	81 (5.7)
Ever incarcerated	262 (19.0)
Incarcerated 6 months or less	253 (17.8)
Incarcerated 6 months or more	97 (7.0)
Number of partners reported on	82 (6.0)
One	405 (28.6)
Two	292 (20.6)
Three	717 (50.6)
Used a condom at last sex^a^	
Yes	1271 (45.0)
No	1551 (55.0)

^a^Partner level

Bivariable GEE models (see Table 2) indicated that those significantly more likely to report condom use at last sex were: single [OR: 1.63 CI 95 %: (1.23, 2.15)], younger [OR: 1.26 CI 95 %: (1.05, 1.50)], had a college degree [OR: 1.60 CI 95 %: (1.19, 2.14)], and attended religious services at least once a month [OR: 1.27 CI 95 %: (1.06, 1.51)]. Conversely, condom use was less likely with main partners [OR: 0.37 CI, 95 %: (0.32, 0.44)], persons who reported being financially or materially dependent on a partner [OR: 0.39 CI 95 %: (0.31, 0.47)], having a partner that was dependent on the participant [OR: 0.34, CI 95 %: (0.28, 0.43)], being female [OR: 0.76 CI 95 %: (0.63, 0.90], heavy episodic drinking less than monthly [OR: 0.77, CI 95 %: (0.60, 0.97)], and a history of cocaine or crack use [OR: 0.52 CI 95 %: (0.33, 0.85)]. With regard to incarceration experience, only having been incarcerated two or more times for more than 6 months was associated with a significant decrease in the likelihood of condom use at last sex [OR = 0.47, 95 % CI = 0.27, 0.82].

**Table 2:** Univariate correlates of event level condom use at last sex reported by participants attending a publicly funded STI clinic in Jackson, Mississippi

Variable	% Used Condom at Last Sex with Partner	Odds ratio (95 % CI)	*P*-value
*Demographics*			
Female			
Yes	42.3	0.76 (0.63, 0.90	0.002
No	48.8		
Age			
24 or younger	47.0	1.26 (1.05, 1.50)	0.011
25 or older	41.1		
Single			
Yes	46.2	1.63 (1.23, 2.15)	0.001
No	35.3		
Sexual Orientation			
Heterosexual	44.9	REF	
Gay/Lesbian	50.9	1.21 (0.73, 1.99)	0.457
Bisexual	43.9	0.39 (0.69, 1.39)	0.914
Education			
High school or less	40.1	REF	
Some college	48.5	1.23 (0.89, 1.69)	0.210
College degree or higher	47.7	1.60 (1.19, 2.14)	0.002
Stable Housing			
Yes	43.5	0.89 (0.75, 1.05)	0.165
No	46.4		
Attends religious services at least once a month			
Yes	47.6	1.27 (1.06, 1.51)	0.008
No	42.0		
Main partner			
Yes	34.2	0.37 (0.32, 0.44)	<0.001
No	57.4		
Depend on partner			
Yes	27.4	0.39 (0.31, 0.47)	<0.001
No	49.3		
Partner depends on participant			
Yes	24.7	0.34 (0.28, 0.43)	<0.001
No	49.7		
*Substance Use & Sexual History*			
Binge drinking			
Never	46.5	REF	
Less than monthly	40.6	0.77 (0.60, 0.97)	0.026
Monthly	46.5	1.00 (0.75, 1.34)	0.975
At least once a week	33.9	0.61 (0.35, 1.04)	0.069
Cocaine/crack use ever			
Yes	34.4	0.52 (0.33, 0.85)	0.008
No	45.7		
Alcohol use at last sex			
Yes	44.2	1.03 (0.82, 1.29)	0.810
No	45.2		
Partner alcohol use at last sex			
Yes	41.2	0.95 (0.77, 1.17)	0.659
No	44.7	REF	
Don’t know	59.9	1.75 (1.27, 2.43)	
Drug use at last sex			
Yes	40.5	0.79 (0.61, 1.03)	0.078
No	45.5		
Partner drug use at last sex			
Yes	38.4	0.83 (0.65, 1.07)	0.148
No	44.9	REF	
Don’t know	59.4	1.60 (1.14, 2.25)	0.006
Lifetime number of sex partners			
1–5	45.3	REF	
6–10	45.7	1.02 (0.81, 1.28)	0.880
> 10	44.3	0.83 (0.64, 1.07)	0.970
Ever had anal sex with partner			
Yes	43.6	0.80 (0.62, 1.03)	0.080
No	47.3		
*Incarceration Experiences*			
Ever incarcerated			
Yes	43.0	0.92 (0.73, 1.13)	0.413
No	45.6		
Incarcerated less than 6 months			
Never	45.5	REF	
Once	42.1	0.90 (0.60, 1.33)	0.597
2 or more times	34.6	0.66 (0.36, 1.21)	0.183
Incarcerated more than 6 months			
Never	45.6		
Once	45.1	0.99 (0.62, 1.56)	0.954
2 or more times	26.3	0.47 (0.27, 0.82)	0.008
Partner incarcerated past 6 months			
Yes	39.5	0.79 (0.57, 1.10)	0.165
No	45.6		

*REF* reference category

The results of the final multivariable GEE logit model are shown in Table 3. Being female [adjusted OR (AOR): 0.71 CI 95 %: (0.58, 0.87)], engaging in heavy episodic drinking less than monthly [AOR: 0.71 CI 95 %: (0.55, 0.92)], having more than 10 lifetime partners [AOR: 0.73 CI 95 %: (0.58, 0.93)], considering their current partner as their main partner [AOR: 0.42 CI 95 %: (0.35, 0.50)], and having a partner who financially or materially depended on them [AOR: 0.50 CI 95 %: (0.40, 0.63)] were all independently associated with a lower likelihood of condom use at last sex. Having some college education and having a college degree were both associated with an increased likelihood of using condoms at last sex [AOR: 1.35 CI 95 %: (1.11, 1.65) and AOR: 1.45 CI 95 %: (1.08, 1.94) for some college education and college degree or higher, respectively]. After adjusting for covariates and other incarceration variables, having been incarcerated two or more times for more than 6 months remained significantly associated with decreased condom use. The odds of using a condom at last sex were 2.3 times lower [AOR: 0.44 CI 95 %: (0.24, 0.81)] for participants who reported having been incarcerated multiple times for more than 6 months.

**Table 3:** Multivariable model results for incarceration correlates of condom use at last sex reported by participants attending a publicly funded STI clinic in Jackson, Mississippi

Variable	Adjusted Odds Ratio (95 % CI)	*P*-value
*Covariates*		
Female	0.71 (0.58, 0.87)	0.001
Education		
High school or less	REF	
Some college	1.35 (1.11, 1.65)	0.004
College degree or higher	1.45 (1.08, 1.94)	0.014
Attends religious service at least once a month	1.18 (0.98, 1.43)	0.075
Main partner	0.42 (0.35, 0.50)	<0.001
Partner depends on participant	0.50 (0.40, 0.63)	<0.001
Heavy episodic drinking		
Never	REF	
Less than monthly	0.71 (0.55, 0.92)	0.008
Monthly	0.96 (0.71, 1.31)	0.802
At least once a week	0.58 (0.32, 1.05)	0.070
Lifetime number of sex partners		
1–5	REF	
6–10	0.90 (0.70, 1.14)	0.374
> 10	0.73 (0.58, 0.93)	0.010
*Incarceration Variables*		
Incarcerated less than 6 months		
Once vs. Never	0.78 (0.42, 1.46)	0.440
2 or more times vs. never	1.73 (0.49, 6.08)	0.391
Incarcerated more than 6 months		
Once vs. Never	1.29 (0.79, 2.12)	0.303
2 or more times vs. never	0.44 (0.24, 0.81)	0.008
Partner incarcerated past 6 months	0.93 (0.65, 1.34)	0.710

*REF* reference category

## Discussion

This study, conducted at a public STI clinic in Jackson, Mississippi, is among the first to explore the relationships between duration of incarceration, multiple incarcerations and condom use. The findings of this study demonstrate that repeated incarceration for more than 6 months at a time was independently associated with increased odds of condomless sex. The current study is also among the first studies to explore incarceration and condom use in the Deep South, which has the highest rates of STIs and HIV in the country [22].

Our findings add to a mounting body of evidence that links incarceration to risky health behaviors and poor health outcomes [23, 24]. This study also supports other recent findings that demonstrate the relationship between incarceration and lower rates of condom use post-release [11, 25]. Reasons for this association might include longer periods of sexual deprivation and lack of access to sexual health education while incarcerated and post-release. The findings of the current study highlight that longer-term and repeated incarceration is associated with condom use while short-term incarceration was not associated with condom use. Previous research has demonstrated that incarceration can dissolve primary sexual relationships, and, thus, is related to increased sexual risk taking after release, which may be especially true for those serving long sentences [18]. In addition, incarceration disrupts existing social support networks and introduces chaos into individuals’ lives in the form of lack of access to economic opportunities and housing, all of which could lead to a de-prioritization of sexual health [24, 26, 27].

Given the association between multiple incarcerations, duration of incarceration and condom use in our study, greater efforts are needed to address the sexual health needs of individuals with criminal justice involvement, especially those with longer and more frequent incarcerations. While many criminal justice systems do provide preventative screenings during incarceration a recent study found that only 46.9 % of all prisons and 16.6 % of jails provided STI testing [28]. Testing initiatives should continue to be strengthened and buttressed with broader sexual health education programs and comprehensive discharge planning that includes linkage to relevant medical care especially for individuals who have previously been incarcerated. Recent research has demonstrated that the effect of expanded HIV testing should be augmented by condom use interventions [29].

In addition, providing incarcerated individuals with discharge planning services that include linkage to sexual health services and provision of condoms upon release could also decrease the likelihood of sexual risk taking in the community. Also Pre-exposure prophylaxis (PrEP) interventions could reduce HIV acquisition risks for HIV-uninfected individuals engaging in condomless sex post-release. PrEP is a once daily medication for at-risk persons that can dramatically reduce the risk of HIV acquisition [30–32]. A recent study found that adherence to PrEP among people who inject drugs to be generally high, but participants who had been incarcerated had lower levels of drug adherence, which dramatically enhances PrEP’s efficacy [33]. Little is currently known about how to implement PrEP after discharge, but the results of the current study suggest that offering PrEP upon release should be explored.

After release STI providers and other community based organizations that care for high-risk patients should adopt tools that screen for criminal justice involvement as a marker of risk. This could take the form of asking about criminal justice history upon intake or during routine appointments or could include cross-sector partnership between criminal justice agencies and community-based clinics. This partnership would facilitate linkage to sexual health services post-release and diminish risk by promoting the use of various STI prevention techniques. With access to history of criminal justice involvement data, providers may be able to better serve the needs of formerly incarcerated patients, tailoring treatment and intervention plans to the potentially enhanced risk introduced by incarceration.

Finally, our findings also have important policy implications. The duration and number of times incarcerated seem to have compounding effects on sexual risk behaviors after release. This finding adds to a growing body of literature that elucidates the negative effects of sentencing policies on correctional and community health. For instance, previous research has shown that incarceration, particularly repeated incarceration, increases the risk of virologic failure among HIV infected people who inject drugs [34, 35]. The rise of the use of mandatory minimum sentencing and “three strikes” laws have resulted in more people, particularly African Americans, becoming incarcerated for longer periods of time [11]. A de-escalation of mass incarceration, which has recently been touted by conservative and liberal policymakers alike, and a move away from harsh sentencing reforms, could have positive benefits for the sexual health of African Americans in the Deep South, who are disproportionately arrested, convicted and incarcerated and tend to serve longer sentences [36].

### Limitations and future research

This study has several limitations. First, the explanatory and outcome variables were based on self-report, which could result in misclassification. In addition, because the study was cross-sectional it is difficult to infer causality between incarceration history and condom use behaviors. Also, the study was limited to persons accessing a publicly funded STI clinic and is also a restricted sample of individuals who have engaged in high-risk sexual behavior. Additionally, the sample is mostly women (nearly 63 %) whereas a much smaller percentage of the justice involved population is female. Because of these sample restrictions generalizability is limited. Moreover, while it is important to know that duration of sentence matters in relation to incarceration and condom use, we only analyzed incarceration of less than 6 months and greater than 6 months due to sample size constraints. Future research should build on our findings and further assess how a wider range of sentence variation affects sexual risk behavior in a larger sample of individuals with a history of incarceration. In addition, we did not ask about type of facility (prison or jail). For instance, jails typically have higher turnover rates than prisons meaning individuals, overall, serve shorter sentences, and, thus, spend less time in jail facilities. Finally, while we found a relationship between longer-term, repeated incarceration and condomless sex, there is still a need to understand the latent factors that undergird this relationship. More information is needed about the mechanisms of both incarceration (e.g. deprivation, isolation) and the post-release experience (e.g. lack of social support) and how they might affect decisions about condom use. Qualitative research would help elucidate how these incarceration-related factors mediate the relationship between incarceration and condom use.

## Conclusion

This study found a strong, independent relationship between condom use and multiple, long-term incarceration events among patients in an urban STI clinic in the Deep South. Efforts to provide comprehensive sexual health, HIV and STI screening services, and PrEP, in combination with increased access to condoms for inmates during incarceration and upon release, could lead to less sexual risk taking post-release. Ultimately, reducing the number of incarcerated individuals could have positive effects on the sexual health of those with criminal justice experience as well as their home communities.
